# Effects of sepsis on respiratory mechanics in a porcine model of intra-abdominal hypertension

**DOI:** 10.1186/cc14468

**Published:** 2015-03-16

**Authors:** B Fyntanidou, K Kotzampasi, M Kyparissa, G Stavrou, E Oloktsidou, X Mpesi, K Papapostolou, V Grosomanidis

**Affiliations:** 1 Aristotle Medical School, Thessaloniki, Greece

## Introduction

The aim of our study was to investigate the effects of sepsis on respiratory mechanics in a porcine model of intra-abdominal hypertension (IAH).

## Methods

Sixteen pigs were divided into two groups of eight (G-A/ G-B). All animals received general anesthesia and were mechanically ventilated. Parameters recorded included respiratory system, chest wall and lung compliance (CRS, CCW, CL) and respiratory system and chest wall inspiratory and expiratory resistances (RRSisp, RRSexp, RCWisp, RCWexp). After baseline measurements (0 minutes), intraabdominal pressure IAP was raised by helium insufflation to 25 mmHg in both groups and remained at that level for the whole study. In G-B, sepsis was induced 60 minutes after IAP increase, by i.v. administration of *Escherichia coli *endotoxin. Parameters were recorded every 20 minutes. The last measurement was made at 180 minutes, right after deinsufflation, and IAP return to baseline levels.

## Results

CRS decreased statistically significantly in both groups after IAP increase and increased after deinsufflation only in G-A. Similarly, CCW decreased in both groups but returned to baseline values in both groups after deinsufflation. CL decreased more significantly in G-B and returned to baseline values only in G-A. RRSisp increased only in G-B and did not decrease after deinsufflation, whereas RRSexp increased in both groups, in a more significant manner in G-B, and decreased only in G-A after deinsufflation. RCWisp and RCSesp did not show any alterations during the study period. Results are depicted as mean values ± SD in Tables 1 and 2.

## Conclusion

Both sepsis and IAH have negative effects on respiratory mechanics. However, their combination has even more detrimental effects, which do not ameliorate after deinsufflation.

**Table 1:** Compliance alterations during the study.

**C_RS_ (ml/cmH_2_O)**				
**C_CW_ (ml/cmH_2_O) **		**C_L_ (ml/cmH_2_O) **		
**Minutes **	**A **	**B **	***P *value **	**A **
**B **	***P *value **	**A **	**B **	***P *value **
0	35 ± 8	31 ± 4	NS	58 ± 20
46.1 ± 11	NS	99 ± 22	98 ± 14	NS
20	14 ± 4^**^	12 ± 3^**^	NS	20 ± 6^**^
17.7 ± 3^**^	NS	52 ± 21^**^	35 ± 11^**^	NS
40	13 ± 4^**^	12 ± 1^**^	NS	19 ± 6^**^
16.8 ± 3^**^	NS	47 ± 23^**^	36 ± 13^**^	NS
60^a^	12 ± 3^**^	11 ± 2^**^	NS	20 ± 5^**^
18.4 ± 4^**^	NS	41 ± 20	28 ± 7^**^	NS
80	13 ± 3^**^	10 ± 1^**^	NS	20 ± 6
18.2 ± 44	NS	41 ± 21^**^	24 ± 6^**^	<0.05
100	12 ± 3^**^	10 ± 2^**^	NS	21 ± 8^**^
19.9 ± 5^**^	NS	37 ± 20^**^	19 ± 4^**^	<0.05
120	12 ± 3^**^	10 ± 1^**^	NS	20 ± 6^**^
21.9 ± 7^**^	NS	37 ± 20^**^	17 ± 3^**^	<0.05
140	12 ± 3^**^	9 ± 1^**^	NS	21 ± 7^**^
21.8 ± 6^**^	NS	37 ± 20^**^	17 ± 5^**^	<0.05
160	12 ± 3^**^	9 ± 1^**^	NS	21 ± 7^**^
22.3 ± 7^**^	NS	37 ± 19^**^	16 ± 4^**^	<0.05
180	33 ± 7^**^	16 ± 5^**^	<0.001	50 ± 18
77.0 ± 18^**^	<0.05	81 ± 20^*^	21 ± 7^**^	<0.001

^a^Sepsis induction. Comparison with baseline: ^*^P < 0.05, ^**^P<0.01.

**Table 2:** Respiratory system resistances alterations during the study.

		**R_RSisp_ (cmH_2_O/l/minute)**	
**R_RSexp_ (cmH_2_O/l/minute)**			
**Minutes**	**A**	**B**	***P***
**value **	**A **	**B**	***P ***
**value **			
0	8.1 ± 0.8	8.3 ± 0.7	NS
13.6 ± 4.1	15.5 ± 3.7	NS	
20	7.8 ± 0.6	8.1 ± 0.7	NS
17.1 ± 5.2^**^	19.3 ± 2.2**	NS	
40	7.8 ± 0.8	7.6 ± 0.9	NS
18.1 ± 5.4**	20.6 ± 3.1**	NS	
60^a^	7.6 ± 0.9	7.5 ± 1.1	NS
18.9 ± 4.4**	19.9 ± 4.7**	NS	
80	7.8 ± 1.2	8.2 ± 1.2	NS
18.6 ± 4.2**	21.1 ± 4.12**	NS	
100	7.8 ± 0.9	7.9 ± 0.9	NS
18.2 ± 4.5**	22.7 ± 4.5**	NS	
120	8 ± 0.6	9.1 ± 1.1	
<0.05	18.6 ± 4.3**	20.9 ± 2.2**	NS
140	7.7 ± 0.6	9.5 ± 1.2	
<0.01	18.8 ± 3.5**	22.9 ± 3.2**	
<0.05			
160	7.3 ± 0.7	9.6 ± 1.5*	
<0.01	17.7 ± 3.5**	22.2 ± 2.3**	
<0.01			
180	8.1 ± 0.8	9.7 ± 1.2*	
<0.01	14.9 ± 3.3	18.9 ± 2.3*	
<0.05			

^a^Sepsis induction. Comparison with baseline: ^*^P < 0.05, ^**^P < 0.01.

